# Mining Database for the Clinical Significance and Prognostic Value of ESRP1 in Cutaneous Malignant Melanoma

**DOI:** 10.1155/2020/4985014

**Published:** 2020-09-05

**Authors:** Baihe Wang, Yang Li, Caixia Kou, Jianfang Sun, Xiulian Xu

**Affiliations:** ^1^Institute of Dermatology, Chinese Academy of Medical Sciences and Peking Union Medical College, 12 Jiangwangmiao Street, Nanjing 210042, China; ^2^Department of Dermatology, The Affiliated Qingdao Municipal Hospital of Qingdao University, Qingdao, China

## Abstract

**Background:**

Epithelial splicing regulatory protein 1 (ESRP1) has been described as an RNA-binding protein involved in cancer development. However, the expression and regulatory network of ESRP1 in cutaneous malignant melanoma (CMM) remain unclear.

**Methods:**

From the sequencing data of 103 CMM samples in The Cancer Genome Atlas database, the expression level of ESRP1 and its correlation with the clinicopathological characteristics were analyzed using the Oncomine 4.5, Gene Expression Profiling Interactive Analysis (GEPIA), and UALCAN tools, while LinkedOmics was used to identify differential gene expression with ESRP1 and to analyze Gene Ontology (GO) and Kyoto Encyclopedia of Genes and Genomes (KEGG) pathways. Gene enrichment analysis examined target networks of kinases, miRNAs, and transcription factors. Finally, TIMER was used to analyze the relationship between ESRP1 and tumor immune cell infiltration.

**Results:**

We found that ESRP1 was lowly expressed in CMM tissues, and a low level of ESRP1 expression correlated with better overall survival. Expression of this gene was linked to functional networks involving the condensed chromosomes, epidermal development, and translation initiation. Functional network analysis suggested that ESRP1 regulated ribosome metabolism, drug metabolism, and chemical carcinogenesis via pathways involving several cancer-related kinases, miRNAs, and transcription factors. Furthermore, our results suggested that ESRP1 played an important role in regulating tumor-associated macrophage polarization, dendritic cell infiltration, Treg cells, and T cell exhaustion.

**Conclusion:**

Our study demonstrates ESRP1 expression, prognostic value, and potential regulatory networks in CMM, thereby shedding light on the clinical significance of ESRP1, and provides a novel biomarker for determining prognosis and immune infiltration in CMM.

## 1. Introduction

Melanoma, a common malignant tumor originating from skin melanocytes, is characterized by high invasiveness [1–3]. According to statistics, there are approximately 200,000 newly diagnosed cases each year [4], and melanoma accounts for 80% of deaths related to cutaneous cancers [5]. In the early stages of melanoma, surgery may be an adequate treatment for patients [6]. However, in the late stages of the disease, patients may develop local or distant metastases with a poor prognosis [7]. Therefore, identifying molecular targets related to tumorigenesis and development is of great significance for the treatment of melanoma.

Epithelial splicing regulatory protein 1 (ESRP1) was previously called RBM35A. The gene is located on chromosome 8q22.1, with a sequence length of 2046 bp and a relative molecular weight of 78 × 10^3^, encoding 682 amino acids. As a member of the hnRNP family, ESRP1 plays a vital role in organ formation, including craniofacial and epidermal development, branching morphogenesis of the lungs, and salivary gland development. Recent studies have found that ESRP1 regulates the alternative splicing of multiple genes, including *CD44*, *CTNND1*, *ENAH*, and *FGFR2*, thereby affecting intercellular adhesion, cytoskeleton, and cell migration [8, 9]. Hence, ESRPs contribute to the loss of cell differentiation, which is one of the underlying mechanisms of tumorigenesis. In fact, studies have shown that in multiple tumor cell lines, such as those of prostate cancer, breast cancer, pancreatic cancer, kidney cancer, and head squamous cell carcinoma, tumor invasion is associated with a low expression of ESRPs [10, 11]. However, the specific role of ESRP1 in cutaneous melanoma remains unclear.

In this study, we aimed to systematically explore the gene expression, prognostic values, immune correlations, and potential functions of ESRP1 in CMM. The correlation between ESRP1 levels, clinical parameters, and tumor immune infiltration was comprehensively analyzed. Moreover, we also explored the prognostic value and functions of ESRP1 in CMM. These findings suggest that ESRP1 plays an important role in the clinical prognosis and immune regulation of CMM.

## 2. Materials and Methods

### 2.1. Oncomine 4.5

Oncomine 4.5 (http://www.oncomine.org) is a large oncogene chip database and integrated data mining platform, containing 715 datasets and 86733 samples that is established for collecting, standardizing, analyzing, and delivering cancer transcriptome data [12]. In the current study, the level of ESRP1 in melanoma was analyzed using Oncomine 4.5, with a *P* value of 0.05, a fold change of 2, and a gene rank in the top 10%.

### 2.2. GEPIA

GEPIA (http://gepia.cancer-pku.cn), a freely available comprehensive web-based tool, analyzes expression data at the transcriptional level with 9,736 tumors and 8,587 normal samples from TCGA and GTEx projects. GEPIA was used to analyze the expression and prognostic value of ESRP1 in melanoma.

### 2.3. UALCAN

UALCAN (http://ualcan.path.uab.edu) is a newly developed interactive web server for facilitating tumor subgroup gene expression analyses based on data from TCGA and MET500 [13]. The correlation between the level of ESRP1 and clinicopathologic features of melanoma was analyzed using UALCAN.

### 2.4. LinkedOmics

LinkedOmics (http://www.linkedomics.org) is a flexible, user-friendly portal providing analysis and comparison of cancer multiomics data across 32 TCGA tumor types [14]. We first explored the correlated significant genes of ESRP1 in 103 TCGA CMM samples using the LinkFinder module. Pearson's correlation coefficient was used to analyze the results, which were graphically presented in volcano plots, heat maps, or scatter plots. Gene set enrichment analysis(GSEA) was performed with a minimum number of genes of 3 and a simulation of 500.

### 2.5. GeneMANIA

GeneMANIA (http://www.genemania.org) is a flexible portal that can analyze the functions of gene lists and find neighboring genes by constructing a protein-protein interaction (PPI) network [15]. GeneMANIA was used to visualize the gene networks and predict the function of genes that GSEA identified as being enriched in melanoma.

### 2.6. TIMER

TIMER (http://www.genemania.org) is an immune infiltrates analysis tool that can provide various analyses with a dataset of 10,897 samples [16]. ESRP1 expression and its correlation with the abundance of immune cells and gene marker expression were evaluated using Spearman's correlation. The gene markers included markers of various immune cells, as referenced in previous studies [17–19]. The estimated statistical significance was analyzed using Spearman's correlation.

## 3. Results

### 3.1. Expression Level of ESRP1 in Patients with CMM

**Figure 1:**
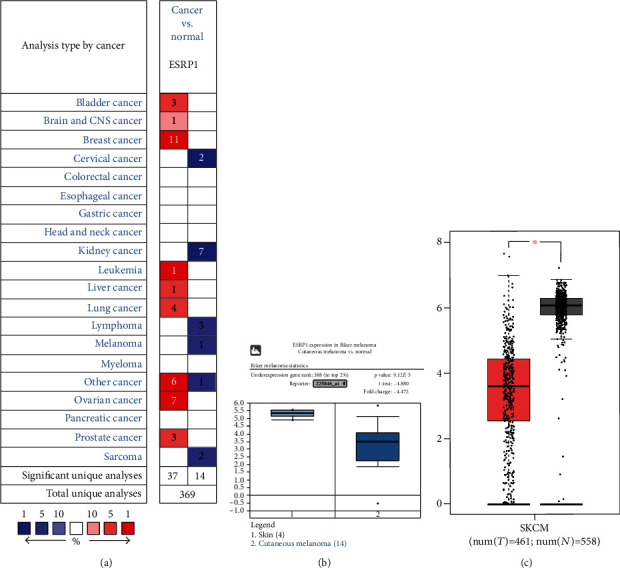
ESRP1 expression level in CMM. (a) Increased or decreased ESRP1 in data sets of different cancers compared to that of normal tissues (Oncomine). (b, c) The expression of ESRP1 was significantly downregulated in the CMM tissue compared to that in the normal tissue (TCGA and GEPIA).

**Figure 2:**
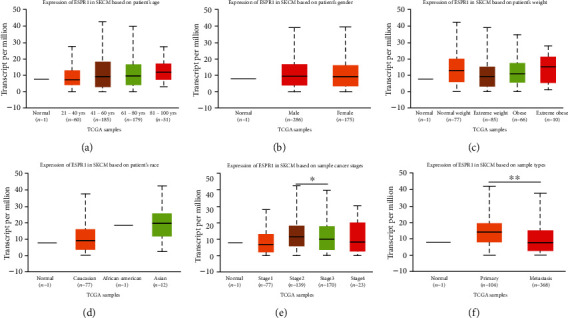
ESRP1 expression level in subgroups of patients with CMM (UALCAN). (a–d) Box plot showing the relative expression of ESRP1 in normal individuals or in different ages, genders, weights, and races of CMM patients (*P* > 0.05). (e) Box plot showing the relative expression of ESRP1 in normal individuals or in CMM patients in stages 1, 2, 3, or 4 (*P* < 0.05). (f) Box plot showing the relative expression of ESRP1 in normal individuals or in primary or metastasis CMM patients (*P* < 0.01). Data are represented as mean ± SE. ^∗^*P* < 0.05; ^∗∗^*P* < 0.01.

The expression of ESRP1 was significantly downregulated in CMM tissues compared to normal tissues, based on the data from Oncomine 4.5 (Figures 1(a) and 1(b), *P* < 0.05). Data from Riker et al. [20] has also revealed that ESRP1 was significantly decreased in CMM tissues with a *P* value of 9.12*E*-5 and a fold change of -4.472 (Figure 1(b)). Moreover, GEPIA data demonstrated a significant downregulation of ESRP1 in CMM tissues (Figure 1(c), *P* < 0.05). We then analyzed the correlation between the level of ESRP1 and clinicopathologic features in melanoma. We found no significant difference in the subgroup analyses by age, gender, weight, and race (Figures 2(a)–2(d)). However, there was a remarkable downregulation of the ESRP1 mRNA expression in subgroup analyses based on tumor stage (Figure 2(e)) and lymph node metastasis status (Figure 2(f)).

### 3.2. Prognostic Value of ESRP1 in Patients with CMM

**Figure 3:**
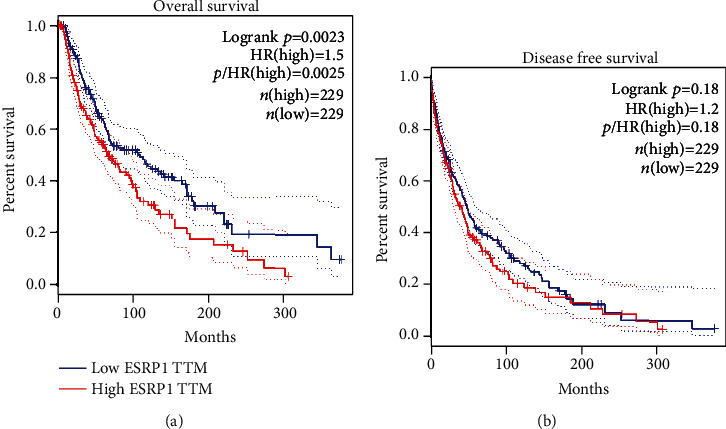
Kaplan-Meier survival curves comparing the high and low expressions of ESRP1 in CMM patients (GEPIA). (a) The overall survival curve for CMM patients with high or low expression of ESRP1 (*P* < 0.01). (b) The disease-free survival curve for CMM patients with high or low expression of ESRP1 (*P* > 0.05).

We also explored the significance of ESRP1 in the prognosis of patients with CMM. Consequently, we found that the CMM patients in the higher ESRP1 level group had poor overall survival, while patients in the low ESRP1 level group had good overall survival (Figure 3(a), *P* = 0.0023). However, there was no significant difference between the high ESRP1 level group and the low ESRP1 level group with regard to disease-free survival (Figure 3(b), *P* = 0.18).

### 3.3. Enrichment Analysis of ESRP1 in CMM

**Figure 4:**
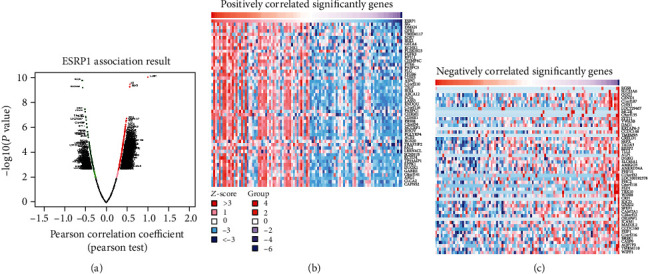
Genes differentially expressed in correlation with ESRP1 in CMM (LinkedOmics). (a) A Pearson test was used to analyze correlations between ESRP1 and genes differentially expressed in CMM. (b, c) Heat maps showing the top 50 significant genes positively and negatively correlated with ESRP1 in CMM. Red indicates positively correlated genes, and green indicates negatively correlated genes.

As shown in Figure 4(a), a positive correlation was obtained between ESRP1 and 788 genes (FDR < 0.05). In contrast, 243 genes (dark green dots) showed a negative correlation with ESRP1 (FDR < 0.05). The top 50 significant genes that positively and negatively correlated with ESRP1 are shown in Figure 4(b) and 4(c), respectively.

**Figure 5:**
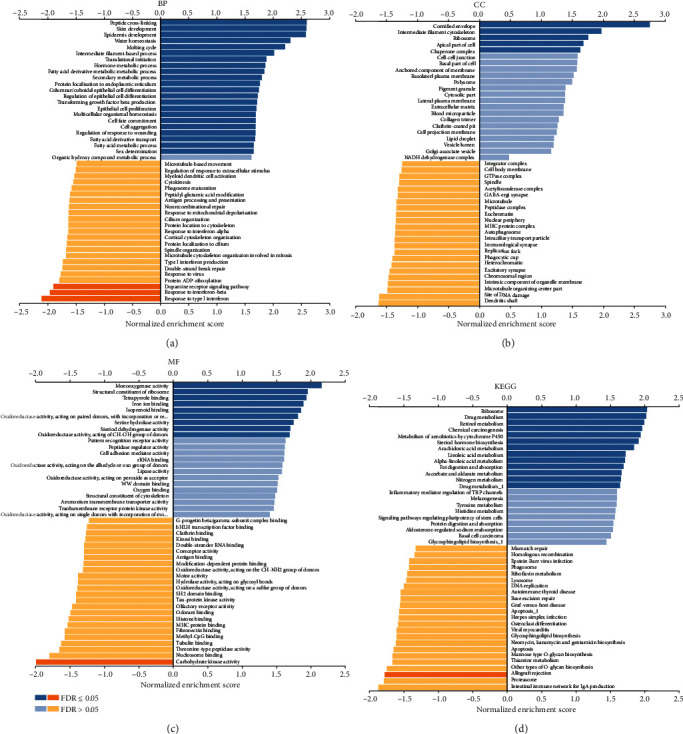
GO annotations and KEGG pathways of ESRP1 in CMM (LinkedOmics). (a) Cellular components. (b) Biological processes. (c) Molecular functions. (d) KEGG pathway analysis.

**Figure 6:**
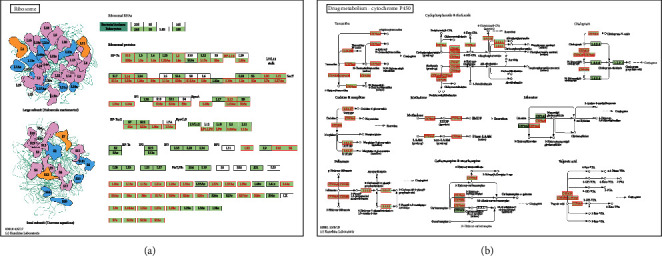
KEGG pathway (LinkedOmics). (a) KEGG pathway annotations of the ribosome metabolism. (b) KEGG pathway annotations of the drug metabolism. Red marked nodes are associated with the leading edge gene.

A strong positive correlation was observed between *ESRP1* and the expression of *XG* (Supplementary Figure 1A, Pearson′s correlation = 0.569, *P* = 3.574*e*–10), *DMKN* (Supplementary Figure 1B, Pearson′s correlation = 0.564, *P* = 5.69*e* − 10), and *GPR1* (Supplementary Figure 1C, Pearson′s correlation = 0.486, *P* = 1.95*e*–07). However, a strong negative correlation was obtained between *ESRP1* and the expression of *RGS8* (Supplementary Figure 1D, Pearson′s correlation = −0.577, *P* = 1.83*e*–10), *SLC22A6* (Supplementary Figure 1E, Pearson′s correlation = −0.562, *P* = 6.69*e* − 10), and *OGG1* (Supplementary Figure 1F, Pearson′s correlation = 0.511, *P* = 3.57*e*–08). GSEA was performed to analyze the GO functional enrichment. The results demonstrated that the expression of ESRP1 is linked to functional networks involving the condensed chromosome, epidermis development, and translational initiation (Figures 5(a)–5(c)). Moreover, functional network analysis suggested that ESRP1 regulates the ribosome, drug metabolism, and chemical carcinogenesis (Figures 5(d), 6(a), and 6(b)).

### 3.4. Kinase, miRNA, and Transcription Factor Target Networks of ESRP1 in CMM

We found that the top 5 significant kinase target networks related to ESRP1 were cyclin-dependent kinase 1 (CDK1), G protein-coupled receptor kinase 3 (GRK3), protein kinase cAMP-activated catalytic subunit beta (PRKACB), protein kinase cAMP-activated catalytic subunit gamma (PRKACG), and protein kinase, X-linked (PRKX) (Table 1). The top 5 miRNA target networks were CACCAGC, miR-138; ATGAAGG, miR-205; GACAATC, miR-219; ACAACCT, miR-453; and ACCGAGC, miR-423 (Table 1). The top 5 transcription factor target networks were mainly associated with the ETF, E2F, EN, USF, and CEBPB transcription factor families (Table 1).

**Figure 7:**
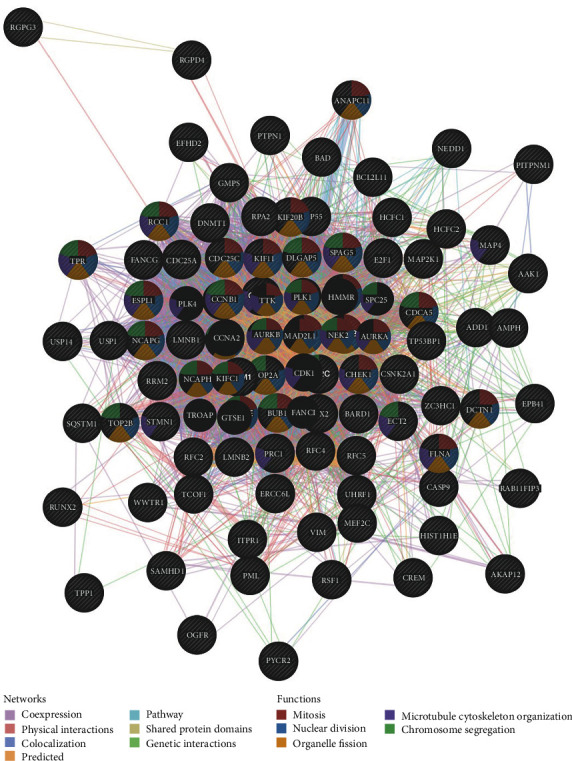
PPI network of CDK1 kinase target networks (GeneMANIA). PPI network and functional analysis indicating the gene set that was enriched in the target network of CDK1 kinases. Different colors of the network edge indicate the bioinformatics methods applied: coexpression, website prediction, pathway, physical interactions, and colocalization. The different colors for the network nodes indicate the biological functions of the set of enrichment genes.

Moreover, GeneMANIA was used to construct a protein-protein interaction (PPI) network to reveal correlations among genes for the kinases CDK1, miRNA-138, and ETF_Q6. As a result, the gene set enriched for kinase CDK1 was involved in the regulation of mitosis, nuclear division, organelle fission, microtubule cytoskeleton organization, and chromosome segregation (Figure 7). The gene set enriched for miR-138 was responsible for the regulation of membrane depolarization, regulation of membrane potential, monovalent inorganic cation transport, monovalent inorganic cation transmembrane transporter activity, and inorganic cation transmembrane transporter activity (Supplementary Figure 2). In addition, the gene set enriched for ETF_Q6 was mainly involved in amino acid regulation, cellular response to amino acid stimulus, negative regulation of intracellular signal transduction, cellular response to acids, TOR signaling, and positive regulation of CREB transcription factor activity (Supplementary Figure 3).

### 3.5. The Potential of ESRP1 as an Immune Biomarker in CMM

**Figure 8:**
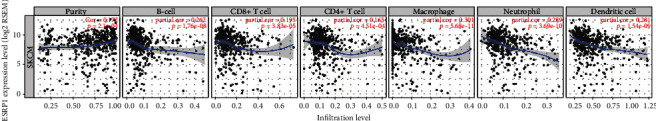
Correlation of ESRP1 expression with immune infiltration level in CMM (TIMER). The expression of ESRP1 was negatively associated with the infiltration abundance of B cells, CD8+ T cells, CD4+ T cells, macrophages, neutrophils, and dendritic cells.

As shown in Figure 8, the expression of ESRP1 was negatively associated with the infiltration abundance of B cells (Cor = −0.262, *P* = 1.76*e* − 08), CD8+ T cells (Cor = −0.195, *P* = 3.83*e* − 05), CD4+ T cells (Cor = −0.165, *P* = 4.51*e* − 04), macrophages (Cor = 0.301, *P* = 5.68*e* − 151), neutrophils (Cor = 0.289, *P* = 3.69*e* − 10), and dendritic cells (DCs; Cor = −0.281, *P* = 1.54*e* − 09).

In order to analyze the potential of ESRP1 as an immune biomarker in CMM, we further analyzed the association between ESRP1 and immune cells. As expected, after adjusting for purity, the data demonstrated a strong association between ESRP1 levels and most immune biomarkers of a variety of immune cells and different T cells in CMM (Table 2).

Specifically, the expression level of ESRP1 was significantly associated with most marker sets of monocytes, TAMs, and M2 macrophages, revealing that ESRP1 may mediate macrophage polarization in CMM. Moreover, low ESRP1 expression is related to high infiltration levels of DCs in CMM. A significant correlation was obtained between ESRP1 expression and expression of DC biomarkers such as HLA-DPB1, BDCA-1, BDCA-4, and CD11c, thus demonstrating a strong relationship between ESRP1 and DC infiltration. In addition, ESRP1 expression negatively correlated with FOXP3, CCR8, STAT5B, and TGFB1 in CMM for Treg cells. Furthermore, ESRP1 expression negatively correlated to PDCD1 (PD-1), CTLA4, LAG3, TIM-3, and GZMB in CMM for T cell exhaustion. Previous studies have demonstrated the significant role of PD-1, CTLA4, and TIM-3 in the immunotherapy of various types of cancers [21–23]. Thus, these results suggested the important role of ESRP1 in tumor immune microenvironment.

## 4. Discussion

CMM is a highly malignant cancer, and metastatic melanoma often leads to a poor prognosis. Nevertheless, its immunogenicity allows intervention via immunotherapeutic strategies such as cytotoxic T lymphocyte-associated antigen 4 (CTLA4) and programmed death-1 (PD-1) inhibitors, which are considered important for the treatment of malignant melanoma [21, 24]. Recently, it has been reported that patients with advanced melanoma achieved a partial response to immunotherapy. Increasing research proved that the levels of tumor-infiltrating lymphocytes (TILs) are associated with response rates to checkpoint blockade in many cancers [25]. Therefore, it is urgent to elucidate the immunophenotypes of tumor-immune interactions as well as immune-related therapeutic targets in CMM.

ESRP1, as a member of an RNA-binding protein family, is an exquisitely epithelial cell-type-specific splicing factor that regulates splicing genes involved in tumor progression [26, 27]. Early onset of an aggressive subgroup of prostate cancer was found to be associated with the expression of ESRP1, indicating that ESRP1 is a potential prognostic marker in prostate cancer [28]. According to Mager LF, the reduced ESRP1 level leads to impaired intestinal barrier integrity, increases susceptibility to colitis, and alters colorectal cancer development [29].

To gain more information about the potential functions of ESRP1 and its regulatory network, we performed target gene analyses of tumor data from public databases. We found that ESRP1 mRNA levels were significantly downregulated in CMM tissues compared to those in normal tissues. Patients with low ESRP1 expression had relatively good overall survival. Related functional networks are involved in epidermal development, translation initiation, ribosome metabolism, drug metabolism, and chemical carcinogenesis. This is consistent with the physiological function of ESRP1 [30]. ESRP1 significantly reduces the growth of tamoxifen-resistant cells and changes epithelial-mesenchymal transition protein markers by affecting metabolic pathways [31], which is consistent with the findings of bioinformatics analysis.

Enrichment analysis found that ESRP1 in CMM is associated with a network of kinases, including CDK1, GRK3, and PRKACB. These kinases regulate mitosis, cell cycle, and cell proliferation [32, 33]. In fact, CDK1 is the main regulator of the cell cycle. CDK1 overexpression in melanoma cells increases carcinogenic potential and tumor initiation ability. Knocking out *Sox2* in CDK1-overexpressed cells can significantly inhibit CDK1; hence, the CDK1-Sox2 interaction is a potential therapeutic target in cancer [34].

miRNAs are small noncoding ribonucleic acid molecules that affect biological processes, including cell proliferation, differentiation, and migration [35, 36]. Our study revealed several miRNAs that were associated with ESRP1, including miRNA-138. Researchers have found that miR-138, miR-155, and miR-221/222 can be used as the diagnostic and prognostic markers of CMM [36–38]. Some studies have reported that miR-138 has tumor-suppressive effects in malignant diseases of the lung, kidney, tongue, head, and neck [39–41].

We found that the top five important transcription factor target networks are ETF, E2F, EN, USF, and CEBPB. ETF, E2F, and SP-1 participate in the cytokine-independent proliferation of mouse hepatocytes [42]. Furthermore, MDM2 relies on the regulation of transcription factor E2F1 to promote the invasion and motility of melanoma cells [43].

Another important aspect of our study is that ESRP1 is negatively related to infiltration of DCs and Treg cells such as FOXP3. It is well known that DCs can promote tumor metastasis by upregulating Treg cells and downregulating CD8+ T cell cytotoxicity [44]. FOXP3 plays a very important role in Treg cells, preventing cytotoxic T cells from attacking tumor cells [24]. Thus, ESRP1 might have the potential to inhibit tumor development by regulating the immunosuppressive microenvironment. Furthermore, in our study, we found that ESRP1 expression was negatively related to T cell exhaustion. T cell exhaustion refers to the loss of functional potential of TILs in the presence of chronic antigens in the tumor microenvironment [45]. Many studies have shown that cellular immune function is decreased when TILs in melanoma tissues express high inhibitory receptors, such as PD-1, CTLA4, and TIM-3 [46–49]. Thus, we speculated that ESRP1 could reflect the immune cell status of tumor patients and could be a predictive target for immunotherapy.

Our study provides a multilevel evidence for the role and potential of ESPR1 as a molecular marker in CMM. However, further studies are required to validate our findings and thus promote the clinical utility of ESRP1 serving as a prognostic indicator or immunotherapy target in CMM.

## 5. Conclusion

In summary, our study highlights the potential utility of ESRP1 status in predicting response to checkpoint blockade immunotherapy and could be a prognosis biomarker in patients with CMM.

## Figures and Tables

**Table 1:** The kinase, miRNA and transcription factor target networks of ESRP1 in CMM (LinkedOmics).

Enriched category	Gene set	Leading edge no.	*P* value
Kinase target	Kinase_CDK1	85	0.0001
	Kinase_GRK3	51	0.0001
	Kinase_PRKACB	28	0.0001
	Kinase_PRKACG	27	0.011
	Kinase_PRKX	27	0.011

miRNA target	CACCAGC, miR-138	45	0.0001
	ATGAAGG, miR-205	38	0.015
	GACAATC, miR-219	26	0.033
	ACAACCT, miR-453	12	0.034
	ACCGAGC, miR-423	3	0.007

Transcription factor target	V$ETF_Q6	48	0.0001
	V$E2F_Q2	32	0.0001
	V$EN1_01	40	0.0001
	V$USF2_Q6	27	0.0001
	V$CEBPB_01	24	0.012

**Table 2:** Correlation analysis between ESRP1 and related genes and biomarkers of immune cells in CMM (TIMER).

Description	Gene markers	CMM			
		None		Purity	
		Cor	*P* value	Cor	*P* value
CD8+ T cell	CD8A	-0.223	^∗∗∗^	-0.143	^∗∗^
	CD8B	-0.201	^∗∗∗^	-0.114	^∗^

T cell (general)	CD3D	-0.215	^∗∗∗^	-0.125	^∗∗^
	CD3E	-0.227	^∗∗∗^	-0.139	^∗∗^
	CD2	-0.226	^∗∗∗^	-0.139	^∗∗^

B cell	CD19	-0.196	^∗∗∗^	-0.128	^∗∗^
	CD79A	-0.185	^∗∗∗^	-0.105	^∗^

Monocyte	CD86	-0.352	^∗∗∗^	-0.272	^∗∗∗^
	CD115(CSF1R)	-0.37	^∗∗∗^	-0.325	^∗∗∗^

TAM	CCL2	-0.357	^∗∗∗^	-0.314	^∗∗∗^
	CD68	-0.179	^∗∗∗^	-0.121	^∗∗^
	IL10	-0.371	^∗∗∗^	-0.325	^∗∗∗^

M1 macrophage	INOS (NOS2)	-0.057	0.219	-0.046	0.326
	IRF5	-0.279	^∗∗∗^	-0.214	^∗∗∗^
	COX2(PTGS2)	-0.292	^∗∗∗^	-0.273	^∗∗∗^

M2 macrophage	CD163	-0.363	^∗∗∗^	-0.317	^∗∗∗^
	VSIG4	-0.346	^∗∗∗^	-0.294	^∗∗∗^
	MS4A4A	-0.326	^∗∗∗^	-0.266	^∗∗∗^

Neutrophils	CD66b (CEACAM8)	-0.042	0.361	-0.054	0.251
	CD11b (ITGAM)	-0.366	^∗∗∗^	-0.317	^∗∗∗^
	CCR7	-0.215	^∗∗∗^	-0.137	^∗∗^

Natural killer cell	KIR2DL1	-0.156	^∗∗^	-0.074	0.113
	KIR2DL3	-0.197	^∗∗∗^	-0.119	^∗^
	KIR2DL4	-0.167	^∗∗∗^	-0.085	0.068
	KIR3DL1	-0.188	^∗∗∗^	-0.11	^∗^
	KIR3DL2	-0.232	^∗∗∗^	-0.152	^∗∗^
	KIR3DL3	-0.046	0.317	-0.012	0.795
	KIR2DS4	-0.12	^∗∗^	-0.052	0.268

Dendritic cell	HLA-DPB1	-0.253	^∗∗∗^	-0.174	^∗∗∗^
	HLA-DQB1	-0.259	^∗∗∗^	-0.191	^∗∗∗^
	HLA-DRA	-0.276	^∗∗∗^	-0.206	^∗∗∗^
	HLA-DPA1	-0.256	^∗∗∗^	-0.186	^∗∗∗^
	BDCA-1(CD1C)	-0.227	^∗∗∗^	-0.161	^∗∗∗^
	BDCA-4(NRP1)	-0.414	^∗∗^	-0.388	^∗∗∗^
	CD11c (ITGAX)	-0.242	^∗∗∗^	-0.171	^∗∗∗^

Th1	T-bet (TBX21)	-0.242	^∗∗∗^	-0.159	^∗∗∗^
	STAT4	-0.204	^∗∗∗^	-0.122	^∗∗^
	STAT1	-0.144	^∗∗^	-0.075	0.111
	IFN-g (IFNG)	-0.22	^∗∗∗^	-0.144	^∗∗^
	TNF-a (TNF)	-0.151	^∗∗^	-0.055	0.241

Th2	GATA3	-0.16	^∗∗∗^	-0.03	0.522
	STAT6	-0.327	^∗∗∗^	-0.341	^∗∗∗^
	STAT5A	-0.206	^∗∗∗^	-0.221	^∗∗∗^
	IL13	-0.057	0.217	-0.023	0.617

Tfh	BCL6	-0.276	^∗∗∗^	-0.27	^∗∗∗^
	IL21	-0.183	^∗∗∗^	-0.132	^∗∗^

Th17	STAT3	-0.196	^∗∗∗^	-0.153	^∗∗^
	IL17A	-0.119	^∗∗^	-0.141	^∗∗^

Treg	FOXP3	-0.226	^∗∗∗^	-0.145	^∗∗^
	CCR8	-0.254	^∗∗∗^	-0.187	^∗∗∗^
	STAT5B	-0.14	^∗∗^	-0.14	^∗∗^
	TGFb (TGFB1)	-0.393	^∗∗∗^	-0.355	^∗∗∗^

T cell exhaustion	PD-1 (PDCD1)	-0.193	^∗∗∗^	-0.103	^∗^
	CTLA4	-0.321	^∗∗∗^	-0.273	^∗∗∗^
	LAG3	-0.206	^∗∗∗^	-0.121	^∗∗^
	TIM-3 (HAVCR2)	-0.331	^∗∗∗^	-0.278	^∗∗∗^
	GZMB	-0.232	^∗∗∗^	-0.147	^∗∗^

## Data Availability

The analyzed data sets generated during the study are available from the corresponding author on reasonable request.

## Supplementary Materials

Supplementary materials Supplementary Figure 1: gene expression correlation analysis for ESRP1 and significant correlated genes (LinkedOmics). The scatter plot shows Pearson's correlation of ESRP1 expression with expression of XG (A), DMKN (B), GPR1 (C), RGS8 (D), SLC22A6 (E), and OGG1 (F). Supplementary Figure 2: PPI network of miR-138 miRNA target networks (GeneMANIA). PPI network and functional analysis indicating the gene set that was enriched in the target network of miR-138. Different colors of the network edge indicate the bioinformatics methods applied: coexpression, website prediction, pathway, physical interactions, and colocalization. The different colors for the network nodes indicate the biological functions of the set of enrichment genes. Supplementary Figure 3: PPI network of ETF_Q6 transcription factor target networks (GeneMANIA). PPI network and functional analysis indicating the gene set that was enriched in the target network of ETF_Q6. Different colors of the network edge indicate the bioinformatics methods applied: coexpression, website prediction, pathway, physical interactions, and colocalization. The different colors for the network nodes indicate the biological functions of the set of enrichment genes. Supplementary Table 1: significantly enriched CDK1 kinase target network of ESRP1 in skin cutaneous melanoma (LinkedOmics). Supplementary Table 2: significantly enriched miR-138 miRNA target networks of ESRP1 in skin cutaneous melanoma (LinkedOmics). Supplementary Table 3: significantly enriched ETF_Q6 transcription factor target networks of ESRP1 in skin cutaneous melanoma (LinkedOmics).
