# Supplementary material for: Mining Database for the Clinical Significance and Prognostic Value of ESRP1 in Cutaneous Malignant Melanoma
Source: Biomed Res Int. 2020 Sep 5;2020:4985014. doi: 10.1155/2020/4985014 (PMC7492958; doi:10.1155/2020/4985014)
Supplement: Supplementary materials — Supplementary Figure 1: gene expression correlation analysis for ESRP1 and significant correlated genes (LinkedOmics). The scatter plot shows Pearson's correlation of ESRP1 expression with expression of XG (A), DMKN (B), GPR1 (C), RGS8 (D), SLC22A6 (E), and OGG1 (F). Supplementary Figure 2: PPI network of miR-138 miRNA target networks (GeneMANIA). PPI network and functional analysis indicating the gene set that was enriched in the target network of miR-138. Different colors of the network edge indicate the bioinformatics methods applied: coexpression, website prediction, pathway, physical interactions, and colocalization. The different colors for the network nodes indicate the biological functions of the set of enrichment genes. Supplementary Figure 3: PPI network of ETF_Q6 transcription factor target networks (GeneMANIA). PPI network and functional analysis indicating the gene set that was enriched in the target network of ETF_Q6. Different colors of the network edge indicate the bioinformatics methods applied: coexpression, website prediction, pathway, physical interactions, and colocalization. The different colors for the network nodes indicate the biological functions of the set of enrichment genes. Supplementary Table 1: significantly enriched CDK1 kinase target network of ESRP1 in skin cutaneous melanoma (LinkedOmics). Supplementary Table 2: significantly enriched miR-138 miRNA target networks of ESRP1 in skin cutaneous melanoma (LinkedOmics). Supplementary Table 3: significantly enriched ETF_Q6 transcription factor target networks of ESRP1 in skin cutaneous melanoma (LinkedOmics). [file 4985014.f1.docx]

**
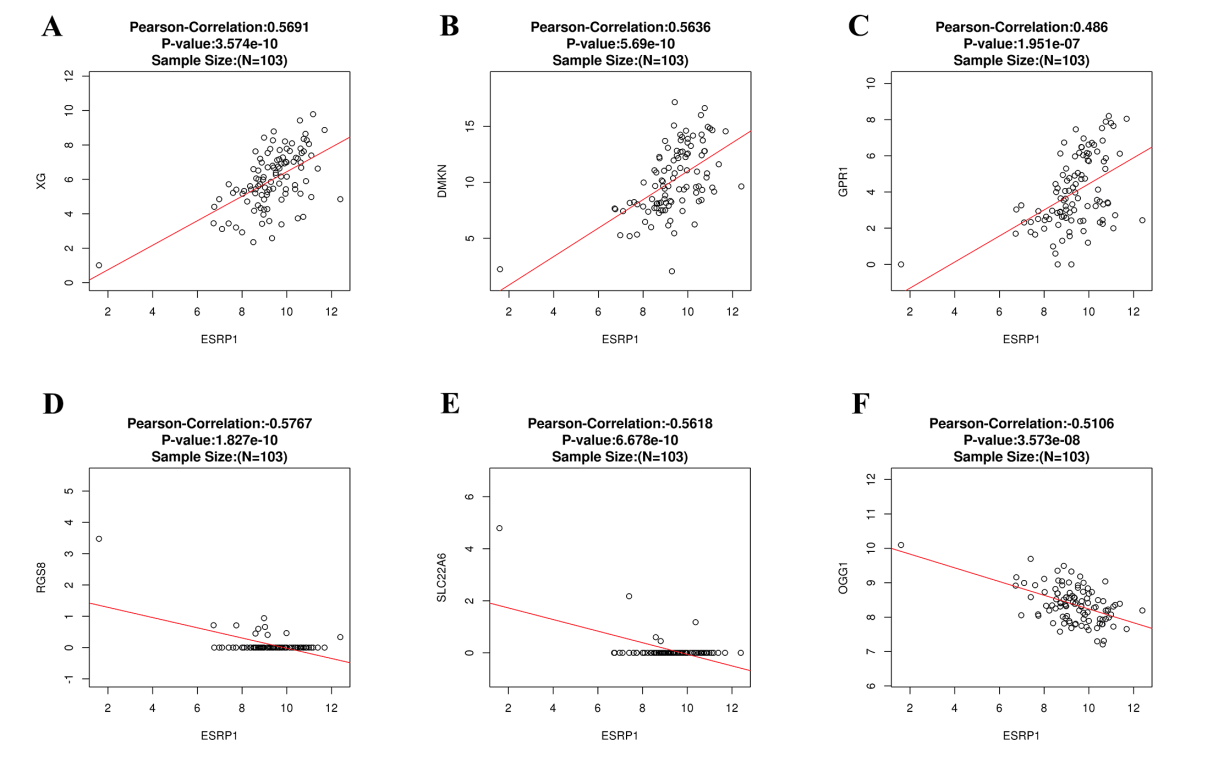
**

**Supplementary Figure 1. *Gene expression correlation analysis for ESRP1 and significant correlated genes (LinkedOmics)*.** The scatter plot shows Pearson correlation of ESRP1 expression with expression of XG (A), DMKN (B), GPR1 (C), RGS8 (D), SLC22A6 (E), and OGG1 (F).


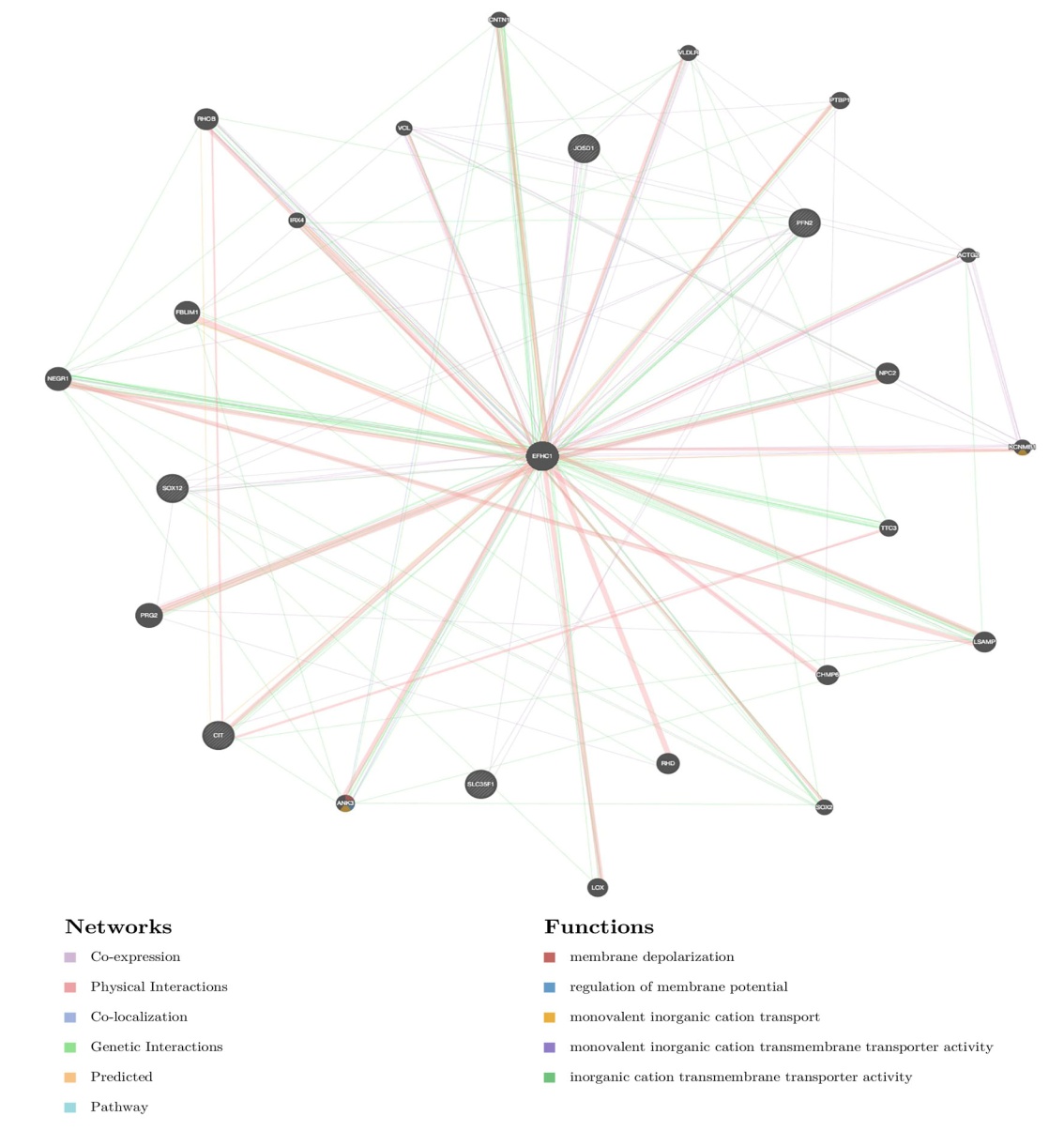


**Supplementary Figure 2.** ***PPI network of miR-138 miRNA-target networks (GeneMANIA).*** PPI network and functional analysis indicating the gene set that was enriched in the target network of miR-138. Different colors of the net-work edge indicate the bioinformatics methods applied: co-expression, website prediction, pathway, physical interactions and co-localization. The different colors for the network nodes indicate the biological functions of the set of enrichment genes.


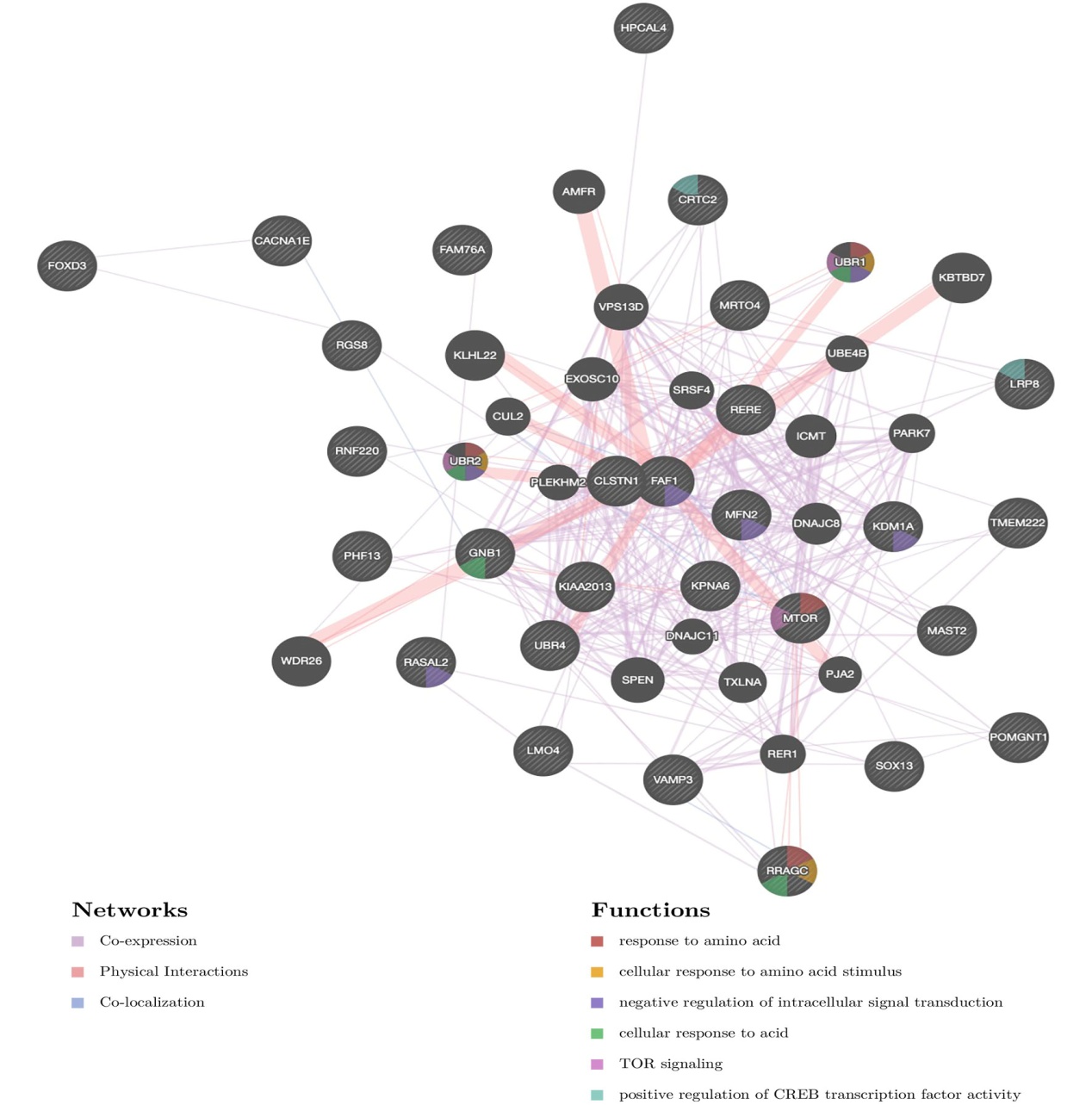


**Supplementary Figure 3.** ***PPI network of*** ***ETF_Q6 transcription factor-target networks (GeneMANIA).*** PPI network and functional analysis indicating the gene set that was enriched in the target network of ETF_Q6. Different colors of the net-work edge indicate the bioinformatics methods applied: co-expression, website prediction, pathway, physical interactions and co-localization. The different colors for the network nodes indicate the biological functions of the set of enrichment genes.
